# Database mining for selection of SNP markers useful in admixture mapping

**DOI:** 10.1186/1756-0381-2-1

**Published:** 2009-02-14

**Authors:** Tesfaye M Baye, Hemant K Tiwari, David B Allison, Rodney C Go

**Affiliations:** 1 Current address: Human and Molecular Genetics Center, Medical College of Wisconsin, 8701 Watertown Plank Road, Milwaukee, WI 53226, USA; 2 Section on Statistical Genetics, Department of Biostatistics, University of Alabama at Birmingham, Birmingham, AL 35294, USA; 3 Clinical Nutrition Research Center, University of Alabama at Birmingham, Birmingham, AL 35294, USA; 4 Department of Nutrition Sciences, University of Alabama at Birmingham, Birmingham, AL 35294, USA; 5 Department of Epidemiology, University of Alabama at Birmingham, Birmingham, AL 35294, USA

## Abstract

**Background:**

New technologies make it possible for the first time to genotype hundreds of thousands of SNPs simultaneously. A wealth of genomic information in the form of publicly available databases is underutilized as a potential resource for uncovering functionally relevant markers underlying complex human traits. Given the huge amount of SNP data available from the annotation of human genetic variation, data mining is a reasonable approach to investigating the number of SNPs that are informative for ancestry information.

**Methods:**

The distribution and density of SNPs across the genome of African and European populations were extensively investigated by using the HapMap, Affymetrix, and Illumina SNP databases. We exploited these resources by mining the data available from each of these databases to prioritize potential candidate SNPs useful for admixture mapping in complex human diseases and traits. Over 4 million SNPs were compared between Africans and Europeans on the basis of a pre-specified recommended allele frequency difference (delta) value of ≥ 0.3.

**Results:**

The method identified 15% of HapMap, 11% of Affymetrix, and 14% of Illumina SNP sets as candidate SNPs, termed ancestry informative markers (AIMs). These AIM panels with assigned rs numbers, allele frequencies in each ethnic group, delta value, and map positions are all posted on our website . All marker information in this data set is freely and publicly available without restriction.

**Conclusion:**

The selected SNP sets represent valuable resources for admixture mapping studies. The overlap between selected AIMs by this single measure of marker informativeness in the different platforms is discussed.

## Background

The chromosome of an individual from a recently admixed population such as the African-American population contains large stretches of DNA that resemble mosaics of chromosomal segments [1], each derived from European or African ancestry that have not had sufficient time to break up as a result of recombination. Hence, allelic associations in these populations may extend over distances as large as 20–30 cM [2,3]. Methods to map genes that rely on admixture may therefore require fewer markers to screen the genome than would other approaches for mapping complex disease genes [4,5].

Theoretically, any marker [6-10] that has an allele frequency difference between ancestral populations, known as ancestry informative markers (AIMs), can be used for admixture mapping. Such markers can also be used to control for population confounding by variations in background ancestry via structural association testing (SAT) [11]. The ideal AIM has one allele that is monomorphic in one population (p = 1.0) and that is not present in another [12]. However, most alleles are shared among populations [13-15]. Hence, it is important to identify and choose informative AIMs across populations [16].

Several single nucleotide polymorphism (SNP) panels have been reported over the past few years [7,8,16-19] with a focus on identifying markers suitable for admixture studies. Smith et al. [9] screened 744 microsatellite markers for AIMs in 4 different populations and identified a genome spanning set of 315 markers (average spacing 10 cM, frequency difference > 0.3) for mapping in African-Americans and 214 markers (average spacing of 16 cM, frequency difference > 0.25) for mapping in Hispanics. Ninety-seven AIMs were identified for mapping in African-American populations that show limited variation within Africa [10].

Recently 3011 SNP AIMs were reported for studying African-American populations [19], who have an average of 80% African and 20% European ancestry, after screening 450,000 SNPs for which allele frequencies were available. This panel is considered the gold standard for admixture mapping in this population. However, the SNPs used to develop these AIMs came mostly from African-American (98.6%, over 443,916 SNPs) populations, and the ancestral West African frequencies were inferred or estimated by using the expectation-maximization (EM) algorithm [20] rather than by being directly measured.

To date, only a limited amount of information characterizing SNPs across the human genome [21,22] for the majority of ethnic groups is found in the literature [23]. Consequently, mining of SNP frequencies from HapMap and other genomic data sets including Affymetrix 500 K and Illumina 100 K SNPs with an ethnic-dependent background across the genome is an economical, rapid, and practical strategy for developing a more comprehensive and informative panel of AIMs [19,24]. This may result in a uniform resource that describes nucleotide diversity with sufficient power to infer ancestry for admixed populations [25], beyond the currently available lists of AIMs. The objectives of the present study were to mine databases and develop AIM panels useful in admixture mapping and compare the selected set of AIMs with the widely used AIM panels.

## Methods

### Materials

SNP markers deposited by the HapMap project, 500 K Affymetrix, 100 K Illumina, and the recently published 3011AIM SNP panels for all autosomal and sex chromosomes were used to determine AIMs. The distribution of SNPs in each chromosome and database is shown in Table 1.

**Table 1:** Number of SNPs investigated for data-mining of AIMs for each chromosome for both Yorubans and European populations listed by genotypic platform or source.

Chr	HapMap	500 k Affymetrix	100 k Illumina	AIMs
1	286584	39418	9820	241
2	304922	40633	8702	230
3	235256	33120	7207	190
4	224433	31339	6000	132
5	230257	31595	6329	136
6	251838	31130	6579	192
7	196235	25407	5581	124
8	199358	26948	4891	129
9	169079	22596	4480	115
10	197292	28217	5240	144
11	189407	28217	5240	144
12	177798	25998	5928	164
13	146641	24712	5465	129
14	114909	18910	3093	76
15	99603	15432	3420	91
16	101959	14190	3307	87
17	83339	15069	3388	103
18	111158	11127	4079	130
19	51689	14631	2570	83
20	111869	6284	3520	117
21	45994	12266	3007	83
22	51037	7014	1381	59
X	103517	6123	1886	94
Y	54	-	-	-

Total	3684228	492556	109366	3011

Chr = chromosome

### Data mining, processing, and description

We downloaded the HapMap allele frequency data (, March 13, 2007 release). The HapMap project contains genotypes from 60 unrelated individuals (parents) from Yorubans in Ibadan, Nigeria [YRI] and 60 unrelated (parents) Caucasians from the United States with northern and western European ancestry [CEU]. There are ~5.8 million SNPs in the combined phase 1 and phase 2 HapMap projects [26,27].

The Affymetrix 500 K was downloaded from . The Affymetrix 500 K array sets contain "quasi-random" or anonymous SNPs that are spread evenly across the genome and are selected on the basis of information content and could lie between genes. These SNPs were developed for genome-wide association and fine mapping studies. The sample sizes used to generate allele frequency datasets of the 500 K SNP array consist of 48 samples containing 13 trios (5 HapMap CEU trios, 5 HapMap Yoruban trios, and 3 other non-HapMap trios) and 9 unrelated HapMap Asian samples. In total, 39 of the 48 samples are parts of the samples used in the HapMap project. About 365,000 or 73% of the Affymetrix 500 K SNPs have also been typed by the HapMap Project.

The Illumina 100 K was downloaded from . This panel is a gene-centric collection of SNPs (70% of which are located in exons or within 10 kb of transcripts) developed for genome-wide association studies. The sample sizes used to generate the Illumina 100 K allele frequency data were 32 CEU and 28 Yorubans. Close to 57,000 of the Illumina 100 K SNPs are in the HapMap project. The recently selected panel of 3011 AIM allele frequencies was obtained from . A total of 66 European Americans and 64 African Americans genotyped by different platforms were used to generate the 3011 AIMs from the total of 450,000 SNPs screened.

Each of these data sets, which differ in the way the SNPs were selected [28], has characteristics that make it useful for the current investigation. The HapMap offers an extensive collection of SNPs across ancestral population genomes; the Affymetrix 500 K is a comprehensive widely used chip; the Illumina 100 K has a gene-centric focus; and the AIM panel is the current gold standard SNP panel used in admixture mapping.

### Data analysis

A computer program using Python  was written to export and pre-process the SNP information from the HapMap databases (the codes are available upon request). A SAS [29] program was used to analyze the data. We used 3 criteria to select the markers to be considered in our analysis: (1) the SNP should be shared between the 2 ancestral populations, (2) a specific marker is retained if it has a delta-value (i.e., the allele frequency difference between 2 parental populations) of 0.3 or higher (a cutoff that has been suggested for AIMs [10], and (3) the physical distance between consecutive selected SNPs must be at least 0.3 cM to avoid the probability of choosing 2 redundant SNPs that are in strong LD [30,31]. It is expected that markers that are sufficiently spaced throughout the genome will offer independent information about genetic background or ancestry. In each of the 0.3 cM bin, AIMs with the highest delta value were selected to maximize information content of ancestry.

Several methods for measuring marker informativeness for ancestry have been developed and discussed by Rosenberg et al. [12] and others [19,32]. However, the absolute allele frequency difference (delta) is the most commonly used measure of informativeness for ancestry between 2 parental populations [12]. Marker informativeness for ancestry can be ascertained through the absolute value of the difference in the frequency of a particular allele observed for 2 ancestral populations. If we let *p*_11 _represent the frequency of a reference allele in the first parental population and *p*_21 _the frequency of the same allele in the second parental population, then the delta value is given by *δ *= |*p*_11 _- *p*_12_|. A marker with a delta value of 1 provides perfect information regarding its ancestry, whereas a marker with a delta value of 0 carries no information for ancestry.

## Results

### 1. SNP allele frequency characterization, racial variation, and databases

Of the total HapMap SNPs for which both Yoruban and CEU allele frequencies were available, we extracted all the monomorphic SNPs and SNPs with various levels of polymorphism, including 100% informative SNPs between the ancestral populations. Table 2 compares the allele frequency distributions under each scenario of the different databases and shows that there is a slight increase in the proportion of rare variation in the Affymetrix and Illumina groups. From the characterized HapMap, Affymetrix, and Illumina SNP databases, 17.3%, 2.6%, and 1.3%, respectively, were 100% noninformative for ancestry.

**Table 2:** Distribution of allele frequency differences (Yoruba vs. European) across SNP marker databases

Allele freq difference	HapMap		Affymetrix		Illumina		AIMs	
	
	SNPs	%	SNPs	%	SNPs	%	SNPs	%
0	635890	17.26	12813	2.60	1392	1.27	-	-
0.01–0.29	2477910	67.257	385585	78.28	91992	84.11	-	-
0.3–0.50	440866	11.966	73066	14.83	15833	14.62	993	33.83
0.51–0.70	114055	3.096	18910	3.84			1515	51.63
0.71–0.90	14957	0.406	2138	0.44	-	-	414	14.11
0.91–0.99	520	0.014	28	0.01	-	-	12	0.40
1	30	0.001	-	-	-	-	-	-

Chr = chromosome

A summary of the interpopulation differences using the HapMap databases shows that a total of only 30 of the interpopulation marker comparisons had very large frequency differences or 100% informative for ancestry (delta = 1) between the 2 ancestral groups (Table 2). The few 100% informative SNPs for ancestry in these findings are consistent with prior studies [33,34], showing that most DNA variation is shared among human populations.

Using a prespecified recommended allele frequency difference (delta) value of ≥ 0.3, on the average across the databases and genome, 15% of HapMap, 19% of Affymetrix, and 15% of Illumina SNP sets were AIMs (Table 3). However, only 15507 (0.42%) HapMap SNPs had an allele frequency difference of 0.7 and above. Similar to the case with CEU, there were large discrepancies in allele frequencies between SNP data for Yoruban populations from the different databases. For example, the reported allele frequencies of the rs55543 SNP from the HapMap, Affymetrix, and Illumina databases were 0.34, 0.31, and 0.42 generated from sample sizes of 120, 48, and 60 samples, respectively. We suspect that the differences in SNP allele frequency data in the different databases were likely due to small sample sizes and respective large sampling errors of the estimates as suggested by Dvornyk et al. [23]. The SNP AIM characteristics with assigned rs numbers, allele frequencies in each ethnic group, delta value, and map positions are all posted on our website . All marker information in this data set is freely and publicly available without restriction.

**Table 3:** Number of AIMs and percentage with delta ≥ 0.3 (in parentheses) for HapMap, Affymetrix, Illumina and AIM databases.

	HapMap SNPs		Affymerix SNPs		Illumina SNPs		AIM SNPs	
				
Chr	Total	delta (%)	Total	delta (%)	Total	delta (%)	Total	delta (%)
1	270009	36255 (13)	39418	4439 (11)	9820	1471 (15)	235	235 (100)
2	293090	46551 (16)	40633	4649 (11)	8702	1280(15)	217	217(100)
3	225937	35394 (16)	33120	3779 (11)	7207	1021(14)	178	178(100)
4	214465	33242 (16)	31339	3449 (11)	6000	929(15)	124	124(100)
5	221858	31821 (14)	31595	3245 (10)	6329	879(14)	129	129(100)
6	244251	32121 (13)	31130	3126 (10)	6579	894(14)	184	184(100)
7	182354	26745 (15)	25407	2785 (11)	5581	826(15)	121	121(100)
8	192846	32106 (17)	26948	3142 (12)	4891	751(15)	122	122(100)
9	162192	23800 (15)	22596	2447 (11)	4480	585(13)	108	108(100)
10	189583	26671 (14)	28217	3005 (11)	5240	784(15)	135	135(100)
11	180434	23850 (13)	28217	2767 (11)	5240	863(15)	154	154(100)
12	169898	23058 (14)	25998	2672 (11)	5928	768(14)	125	125(100)
13	142568	18327 (13)	24712	1909 (10)	5465	399(13)	71	71(100)
14	110229	16581 (15)	18910	1667 (11)	3093	499(15)	88	88(100)
15	95436	16511 (17)	15432	1778 (13)	3420	533(16)	83	83(100)
16	96742	14331 (15)	14190	1741(12)	3307	536(16)	95	95(100)
17	79038	12212 (16)	15069	1342(12)	3388	626(15)	115	115(100)
18	107243	15605 (15)	11127	1613(11)	4079	354(14)	79	79(100)
19	48447	6970 (14)	14631	620(10)	2570	439(12)	102	102(100)
20	108979	12941 (12)	6284	1357(11)	3520	431(14)	80	80(100)
21	43739	6775 (16)	12266	772(11)	3007	169(12)	54	54(100)
22	49009	6419 (13)	7014	620(10)	1381	304(16)	88	88(100)
X	102866	23368 (23)	6123	1867(18)	1886	640(18)	156	156(100)
Y	55	11 (20)	-	-	-	-	-	-

Chr = chromosome

### 2. Number of overlapping AIMs selected from different platforms

We compared selected AIM lists from among the different databases. Even though 57,000 Illumina 100 K SNPs are in the HapMap dataset, there were no common SNPs selected as AIMs when we used a 0.3 and above delta threshold of informativeness. Interestingly, the recent 3011 AIM panel [19] developed from databases such as Applied Biosystems, Applera, SeatleSNPs, and dbSNP is well represented in HapMap (total of 1479 SNPs were common with HapMap as AIMs). Affymetrix 500 K and HapMap have about 365,000 common SNPs. However, for AIMs with a delta value of ≥ 0.3, there were only 26,388 sets of SNPs overlapping between the 2 databases. As AIMs, few SNPs appeared in both Affymetrix and the recently developed AIM panel; the same was true for HapMap, Affymetrix, and the recently developed AIM panel.

However, there was no overlap in the selected AIMs among Affymetrix, Illumina, HapMap, and the recently developed AIM panel (Table 4). This is not surprising because the SNP selection criteria for each platform differed. For example, Affymetrix SNPs are based on proximity to a restriction site and even distribution across the genome, whereas the Illumina platform SNPs are selected in gene-rich regions and thus are not evenly distributed across the genome [28]. Combining nonoverlapping SNPs from different platforms seems a viable approach to increase power and detect signals across the genome.

**Table 4:** Number of overlapping SNP AIMs selected by different platforms (HapMap, Affymetrix, Illumina, and AIMs).

Chr	H<->A	H<->I	H<->S	A <->I	A<->S	I<->S	H<->A<->I	H<-> A<>S	H<->I <->S	A<->I <->S	All
1	1923	0	107	0	5	0	0	1	0	0	0
2	2180	0	114	0	7	0	0	5	0	0	0
3	1983	0	88	0	1	0	0	1	0	0	0
4	1433	0	58	0	6	0	0	3	0	0	0
5	1590	0	71	0	7	0	0	4	0	0	0
6	1836	0	105	0	8	0	0	7	0	0	0
7	1240	0	55	0	2	0	0	2	0	0	0
8	1546	0	61	0	5	0	0	4	0	0	0
9	1073	0	63	0	2	0	0	2	0	0	0
10	1275	0	69	0	5	0	0	2	0	0	0
11	1167	0	67	0	2	0	0	1	0	0	0
12	1071	0	67	0	6	0	0	5	0	0	0
13	1073	0	38	0	1	0	0	1	0	0	0
14	760	0	46	0	2	0	0	2	0	0	0
15	863	0	45	0	2	0	0	2	0	0	0
16	824	0	52	0	4	0	0	3	0	0	0
17	694	0	56	0	2	0	0	2	0	0	0
18	883	0	55	0	5	0	0	3	0	0	0
19	244	0	48	0	1	0	0	1	0	0	0
20	863	0	47	0	3	0	0	3	0	0	0
21	431	0	31	0	6	0	0	5	0	0	0
22	361	0	41	0	5	0	0	1	0	0	0
X	1075	0	95	0	7	0	0	5	0	0	0

Chr = chromosome, H = HapMap, A = Affymerix, I = Illumina, and S = AIMs identified by Smith et al. (2004)

However, most SNPs are not fixed among ancestral populations and so we cannot rule out the chance that the delta measures of informativeness pick different markers in the different platforms. Moreover, the average sample size (number of individuals) or DNA samples in each of the 2 populations used to estimate allele frequencies and the laboratory procedures used vary between platforms. For instance HapMap data were based on 120 samples, Affymetrix was based on 48 samples, and Illumina used 60 samples. Hence, we believe that the selected SNPs that are present in at least 2 platforms could be considered to be the best candidates for admixture mapping.

### 3. Private SNP data set

We observed significant differences in allele frequencies of few SNPs in the present study. These SNPs with significant variation in allele frequencies in populations of different ethnicity may be appropriate for studying the genetic basis of between-ethnic differences in the rates of complex diseases. Although the small sample sizes in this study preclude any definite conclusion regarding the complete absence of a particular allele in any given population, we observed 30 HapMap SNPs (0.001%) that were segregating in only one population sample ("private SNPs"). Most of these private SNPs (77%) were segregating in the African sample, although private SNPs were also observed for European populations. This may owe to the fact that African populations harbor more unique polymorphic alleles than non-African populations [35]. Follow-up studies of the highly differentiated regions might provide significant insight into phenotypic diversity, selection and local adaptation between populations. No private SNPs were observed in the Affymetrix and Illumina data sets.

## Discussion

The SNP databases are important resources for performing genetic linkage, association, and admixture studies. Both academic and commercial groups are developing large numbers of genome-wide SNP datasets. These databases now contain over 12.6 million SNPs. However, only a small fraction of these SNPs are well characterized and validated [21]. Users of these data sets have several common questions regarding the existing databases, including the following: What is the frequency spectrum of the SNPs in these databases? What is the distribution picture of these SNPs across different ethnic and geographic populations? What fraction of the total number of SNPs is already captured by these databases?

We mined and compared the HapMap SNP database against Affymetrix 500 K and the gene centric Illumina 100 K SNP chips. This comparison suggests that a relatively large fraction (> 80%) of SNPs in these databases do not meet the cutoff for acceptable markers as AIMs [10], which means that they are either of very low frequency or not ancestry informative between the 2 ancestral populations. As a result, we developed and preset the AIM panels for each database individually. Our analyses showed that the SNP databases in their current status might have some limitation for studies of complex disorders, especially in different ethnic groups, as a result of incomplete or uneven representation of SNPs along the genome [23]. As indicated above, the different databases have different sets of SNPs. Because the SNP allele frequencies were determined by different genotyping labs that used different sample sizes and genotyping methods (see Methods), it would be difficult to perform several tests to assess data quality and identify sources of experimental variation. In critically evaluating our results, it is important to note that our analyses, and hence interpretations, are subject to several limitations. First, many of our analyses relied on data derived from available databases with contents that are, and will continue to be for some time, in a state of change. Moreover, the allele frequencies across the platforms were based on different sets of DNA samples. Therefore, our results represent a snapshot based on currently available data, and ultimately, when the human genome annotation becomes more stable, it will be important to verify these results. Second, the SNP allele frequencies were determined by using relatively small sample sizes (see Methods), and stochastic variation could affect the robustness of our conclusions.

Several studies discussed the similarities between human populations in terms of genetic constituents, and hence a large sample size may enable the detection of small differences in rare outcomes. Although we observed a strong correlation in allele frequencies between SNPs from different platforms (data not shown), confirming these allele frequency estimates in a larger sample size will be important. The analytical caveats associated with each database, such as how surrogates are Yorubans or CEU to each ancestral population and how much of the data (for example, in HapMap) is transferable to the diverse populations in Africa where there is extreme adaptive variation along the various countries is also debatable.

Most studies consider Europe as a relatively homogeneous population. Consequently, it has been argued that European population stratification does not represent a substantial source of bias in epidemiologic studies [36]. However, recent autosomal SNP studies have highlighted significant patterns of structure within Europe along a north-south axis [37] and also the presence of several significant axes of stratification within Europe, most prominently in a northern-southeastern trend, but also along an east-west axis. The study emphasized the importance of considering population stratification in studies using European and European-American individuals, and the need to develop EuroAIMs (European ancestry informative markers) for ancestry estimation and correction [38]. Moreover, the fundamental theorem underpinning HapMap is the common disease common variance (CD/CV) hypothesis [39]. How much information we can capture from rare variants is not clear [40].

## Conclusion

We presented AIM databases for all SNPs that show promise in distinguishing ancestral populations and thus that will be useful in admixture mapping for finding loci influencing complex phenotypes. These databases will also be useful for controlling stratification (or confounding factors) when the variation in admixture levels among individuals causes false-positive associations in genetic association studies. This investment will result in a unique genetic resource of high quality and global importance for genetic studies in admixed populations. Its size and complexity will allow systematic research into the genetics of many complex disorders in admixed populations and thus, by serving a wide variety of disciplines, will feed research in this promising area for many years to come.

## Competing interests

The authors declare that they have no competing interests.

## Authors' contributions

TMB and RCG conceived the study, and TMB carried out the data mining approaches and drafted the manuscript. HKT and DBA critically commented on the manuscript. All authors read and approved the final manuscript.
